# Spinal cord injury due to road traffic accident in the pre-hospital phase: a grounded theory study in an Iranian context

**DOI:** 10.3389/fpubh.2024.1353342

**Published:** 2024-09-04

**Authors:** Elham Sepahvand, Hamidreza Khankeh, Mohammadali Hosseini, Behnam Akhbari

**Affiliations:** ^1^Department of Nursing, School of Nursing and Midwifery, Social Determinants of Health Research Center, Lorestan University of Medical Sciences, Khorramabad, Iran; ^2^Department of Nursing, University of Social Welfare and Rehabilitation Sciences, Tehran, Iran; ^3^Research Center of Health in Emergency and Disasters, University of Social Welfare and Rehabilitation Sciences, Tehran, Iran; ^4^Department of Clinical Science and Education, Karolinska Institutet, Södersjukhuset, Stockholm, Sweden; ^5^Department of Physiotherapy, University of Social Welfare and Rehabilitation Sciences, Tehran, Iran

**Keywords:** spinal cord injury, grounded theory, emergency care, qualitative study, spinal cord

## Abstract

**Introduction:**

Spinal cord injury is a devastating outcome for individuals and a major public health problem that leads to sensory, motor, and autonomic dysfunction and permanent disabilities. Thus, it is necessary to identify the causes of disability and injury both in the accident phase and in the post-accident phase. This study aimed to develop a theory based on which this complex environment can be discovered.

**Methods:**

This research was a grounded theory study with the constant comparative analysis recommended by Corbin and Strauss in 2015. Participants in this study included 24 Participants were selected from Rofideh Rehabilitation Hospital and Shahid Jalaeipour Spinal Cord Injury Center of Tehran city in 2020. A semi-structured interview with an interview guide was used for data collection. Purposeful sampling method was performed within 10 months until data saturation. Lincoln and Guba’s criteria were used to assess the scientific accuracy and validity of the study.

**Findings:**

The results of interviews showed that “uncertainty” was identified as the most important concern of the injured people, and “trying to save the injured” was identified as the most important concern of the witnesses and families of the injured people. The main categories included “emotional interaction,” “overwhelming anxiety,” “the scene shock,” “misunderstanding of the delay,” “inadequate emergency service,” and “insufficient understanding of the injury.”

**Conclusion:**

In a traffic accident, uncertainty about the situation is the main concern of everyone at the crash scene, from pre-hospital emergency personnel, traffic police, and law enforcement officer to the patient’s companions and other witnesses. Further research is needed to shed more light on this issue.

## Introduction

Road Traffic Accident (RTA) injuries are still considered a major public health problem and one of the leading causes of death for people aged 5–29 years old, especially in low and middle-income countries (LMIC). According to global statistics, about 1.35 million deaths and 20–50 million disabilities occur each year following RTAs (1). The World Health Organization (WHO) has ranked RTA as the eighth leading cause of disability. The economic cost of trauma for over 300 million years of healthy life and 11% of Years of Life Lost due to Disability (YLDs) worldwide has been estimated to be $ 518 billion (2).

Road Traffic Accidents cause numerous traumas to the injured. Among the various injuries, injuries to the central nervous system, including brain and spinal cord injuries, are significant due to the severity of the injury and its long-term effects (3). Spinal cord injury is a devastating outcome for individuals and a major public health problem that leads to sensory, motor, and autonomic dysfunction and permanent disabilities (4). Approximately, half of all spinal cord injuries result in neurological defects, which are often severe and sometimes fatal (5).

Patients with spinal cord injuries experience psychosocial problems such as unemployment, inability to perform daily activities, dependence on others, low presence in society and decreased social communication, lack of self-confidence, depression, and suicidal thoughts (6). Changes in appearance, urine and stool incontinence, dependence on others, and the use of mobility aids change one’s mental image of him/herself and consequently impair his/her mental health (7). On the other hand, due to the chronic nature of the disability, the quality of life in these people is mainly reduced compared to healthy individuals. Losing contact with friends and family, reduced social roles, not participating in volunteer activities, changed socioeconomic status, reduced physical health, and mobility limitations can increase social loneliness and isolation, all of which affect the quality of life of people with spinal cord injury (8).

### Spinal cord injury in an accident scene

To reduce these consequences and lower the costs of disability caused by spinal cord injury, it is necessary to identify the causes of disability and injury both in the prevention phase and the post-accident phase (9). A study by Haghparast et al. on the factors affecting the length of hospitalization following a traffic accident has shown that in LMICs, a significant proportion of deaths and disabilities caused by RTAs occur in the post-accident phase (10). Most of the mortalities occur at the time of the accident and during the transfer of an injured patient to a hospital, which shows the importance of care at the crash site and during hospital transfer (11). Thus, the initial management of an injured patient with potential spinal cord injury begins at the crash site. The purpose of pre-hospital management in patients with spinal cord injuries is to reduce neurological injuries and prevent any secondary damage to the neurological system (12).

In Iran, pre-hospital services are provided by emergency medical technicians and nurses under the supervision of a physician (13). The care delivery model in Iran is based on the Angelo-American model. In this system, trained personnel provide primary care to patients at the crash site and then transport the injured person to the hospital with the help of equipment (12). Protocols have been established for pre-hospital care of patients with spinal cord injury, including opening the airway, controlling bleeding, and releasing and fixating the injured person (14).

However, despite these protocols, the incidence of spinal cord injuries due to traffic accidents is still high. Quantitative studies on the provision of care by pre-hospital nurses show that the provision of pre-hospital care is inadequate in developing countries (15). In these countries, a small number of casualties receive primary care at the crash site, and most of them are not even transferred to the hospital properly and safely by ambulance. Injured patients are mainly transferred by relatives, untrained laypeople, and drivers of heavy vehicles (10). It is also estimated that 3–25% of spinal cord injuries occur after or during the patient’s transfer to the hospital. Numerous cases of poor outcomes following patient transfer have been reported (16).

Studies have focused on this issue from a more objective perspective and have examined the quantitative dimensions and mechanism of an accident. Moreover, studies have explored the barriers to care delivery in the pre-hospital phase in our country, but no specific study has been done on potential spinal cord injuries. On the other hand, factors that affect the incidence of spinal cord injury have not been presented in the form of a theory that shows a clear picture of the factors involved, underlying factors, and the consequences of spinal cord injury. It is obvious that no intervention can be implemented to reduce the injury or increase the quality of life of patients with spinal cord injuries unless this complexity is understood. Therefore, understanding the interactions between staff, bystanders and injured people at the crash scene in a specific cultural context can help to improve the pre-hospital care system to similar situations in some extent. Accordingly, this study was conducted to develop a theory based on which this complex environment can be discovered and the services and quality of care for injured patients can be improved.

## Methods

### Study aim and design

This qualitative study was conducted using the grounded theory approach recommended by Corbin and Strauss in 2015. Participants in this study included 24. Participants were selected from Rofideh Rehabilitation Hospital and Shahid Jalaeipour Spinal Cord Injury Center of Tehran city in 2020. The experiences of the researcher as a nurse and a clinical lecturer, his familiarity with the study setting, and his access to knowledgeable and experienced people were the reasons for choosing these centers.

### Participants

The main participants in this study were the injured people being disabled in traffic accidents and the emergency medical personnel. Further, based on the research needs and theoretical sampling, other participants with different experiences (their families, laypeople, rescue forces including the police, and the Red Crescent) were added to the study sample. Hence, participants in this study included 24 people. In the course of the study, in order to increase the validity of the data, two people who had the experience of being transported during traffic accidents but were not disabled were also recruited.

### Sampling

In this study, the purposeful following by theoretical sampling was used to select the participants. In order to consider the maximum variation in the sampling process, an attempt was made to select patients with spinal cord injuries who had different experiences. Sampling was performed for 10 months until data saturation was achieved. Semi-structured interviews using guide questions were used to collect data. Each interview lasted 30–45 min, depending on the participant’s willingness to share their experiences. Before beginning the interviews, informed consent was obtained from every participant. The researcher first explained the research method and purpose to the participants. Then, if they were willing to participate in the study, he arranged the interview time and place with them after obtaining written informed consent from them. Interviews were recorded with a tape recorder. Data saturation occurred in the 24th interview. Participants were asked questions such as “Please describe everything from the time of the accident to the time of arrival in the hospital.” Follow-up questions were also asked for more understanding of the situation, including “What do you mean by that?” or “Can you explain more?”

In the end, the participants were asked to say whatever they wanted to add. Based on the participants’ responses and the interview process, the consequent questions were designed. In addition, the researcher used other methods of data collection, including focus group interviews, which took about 40 min on average.

### Data analysis and theorizing

In the study, the constant comparative analysis recommended by Corbin and Strauss (17) was used for data analysis. Constant comparison in qualitative research is closely related to grounded theory methodology that used for collecting data and analyzing that raw data throughout the research process. In this procedure, In this method, instead of starting with a hypothesis, the research starts using constant comparison and allows for the emergence of concepts and connections during data collection. The researcher continuously collected data, analyze it, and uses what is obtained to collect more data. This ongoing interaction between data collection and analysis ensures that emerging theory is deeply rooted in the data itself. In this method, the data are broken and coded, and by comparing the codes, similarities, and differences are found (17). In this study, constant comparative analysis was performed through the three steps of open, axial, and selective coding. Open coding to develop concepts in terms of their properties and dimensions, and analyzing data for context was performed by reading interview transcripts sentence by sentence to determine the concepts by the first and second authors. Each interview was read and reviewed several times, and after gaining a general understanding, data analysis was performed using an inductive approach (without the researcher’s preconceived notions). Important paragraphs were selected by reading the text verbatim and were underlined to distinguish them from other parts. Axial coding was then performed to categorize the extracted codes that had common concepts. Then, the initial codes or level 1, level 2, and level 3 concepts were created. The main categories were formed by merging subcategories with similar concepts. Data collection and data analysis were simultaneously done for context and process. Memo writing was done as one of the main techniques in the analysis of the present study. Constant data analysis was used by going back and forth between the data, the results of the analysis, and the memos. For ease of data management, MAXQDA software was also used.

### Rigor/trustworthiness

Guba and Lincoln’s criteria were used to assess the trustworthiness of data (18). In order to assess the trustworthiness of data, measures such as long-term engagement with the participants to gain their trust, use of the interview guide, allocation of enough time to conduct interviews, continuous review and constant comparison of data and categories in terms of similarities and differences, review of the findings with participants, and detailed description of data analysis and study process for future readers were taken. In order to comply with the principles of ethics in research, after obtaining permission from the University’s Ethics Committee (IR.USWR.REC.1395.399), the researcher obtained informed consent from the participants before each interview. The participants were also informed that their personal information would remain confidential, they could withdraw from the study at any time, and their participation in the study would be voluntary.

## Findings

In the study, first, the participants’ demographic information was obtained (Table 1), and then, data analysis was performed according to the method recommended by Corbin and Strauss (17).

**Table 1:** Participants’ demographic information.

Row	Participant	Age	Gender	Type of experience	Interview duration	Interview location
1	C1	32	Male	Injured people	35	Rehabilitation Hospital
2	C2	18	Male	Injured people	30	Rehabilitation Hospital
3	C3	50	Male	Injured people	35	Rehabilitation Hospital
4	C4	22	Male	Injured people	30	Rehabilitation Hospital
5	C5	22	Female	Injured people	20	Molavi Rehabilitation Center
6	C6	36	Female	Injured people	40	Molavi Rehabilitation Center
7	C7	19	Female	Injured people	30	Molavi Rehabilitation Center
8	H1	28	Male	Family of injured people	30	Molavi Rehabilitation Center
9	H2	50	Female	Family of injured people	25	Molavi Rehabilitation Center
10	H3	28	Female	Family of injured people	26	Rehabilitation Hospital
11	H4	26	Male	Family of injured people	30	Molavi Rehabilitation Center
12	C8	32	Male	Injured people	34	Rehabilitation Hospital
13	C9	20	Male	Injured people	20	Rehabilitation Hospital
14	C10	35	Male	Injured people	40	Rehabilitation Hospital
15	P1	37	Male	Police	30	Home
16	P2	26	Male	Police	20	Home
17	L1	32	Male	Laypeople	20	Home
18	L2	28	Female	Laypeople	30	Home
19	T1	36	Male	Technician	35	Rehabilitation Hospital
20	T2	35	Male	Technician	40	Rehabilitation Hospital
21	T3	37	Male	Technician	40	Rehabilitation Hospital
22	T4	31	Male	Technician	30	Home
23	T5	25	Male	Technician	25	Home
24	T6	35	Male	Technician	35	Rehabilitation Hospital

The participants in this study were selected based on maximum variation, using purposeful and then theoretical sampling methods. A total of 24 people participated in this study, 10 of whom had spinal cord injuries, two were laypeople, four were families of injured patients, two were police officers, and six were emergency medical technicians who had experience in caring for people injured in such accidents.

A total of 1,384 codes were derived from data analysis, which as later compared and classified into the main categories and sub-categories that changed over time. The obtained concepts were classified into 125 initial codes, 13 subcategories, and four categories. The main categories included emotional interaction, scene shock, insufficient understanding of the injury, and misunderstanding of the delay (Table 2).

**Table 2:** Categories, subcategories, and primary codes.

Categories	Subcategories	Primary code
Emotional interaction	Unplanned interventions	Unprotected release
		Unprotected transmission
		Failure to stabilize the injured
		Removing the injured from the scene of the accident
	Emotional behaviors	Violence against emergency personnel
		Insistence in pulling out the injured
		Insist on a quick rescue
		Emotional dissonance
		Insist on transfer
	Emotional decisions	Haste
		Failure to call the emergency room
		Transporting the injured by private vehicles
		Action for non-secure transfer
	Emotional intervention	Interference in the work of rescuers
		Aid participation without permission
		Gathering at the scene and blocking the path
		Intervention with a sense of savior
Scene shock	Try to saving	Fear of secondary incidents
		Requirement to save
		Instability of the position
		Blame yourself
	Desperation	Injured unrest
		Emergency rescue from the injured
		Deterioration of the injured
	Emotional atmosphere	Inability to manage emotions
		Psychological pressure of laypeople
		Relief excitement
Insufficient understanding of the injury	Deficiency of relief knowledge	Lack of familiarity with proper care
		Lack of familiarity with the principles of transportation
		Lack of first aid knowledge
	Lack of basic medical knowledge	Lack of understanding of spinal cord injury
		Lack of awareness of the function of the spinal cord
		Not having knowledge of the clinical conditions of the injured
		Lack of knowledge of the nervous system
	Lack of comprehensiveness of review	Surface assessment
		Not paying attention to the main problem
		Gradual awareness of the problem
		Failure to perform triage
Misunderstanding of the delay	Time pressure	Fear of death of the injured
		Confusion
		Delay in calling the emergency room
		Lack of awareness of the situation
		Lack of awareness of time
	Previous experiences of companions	Ambulance delay experience
		Experience of the death of loved ones in accidents
		Experience of similar scenes
	Failure to attend the emergency room on time	Obligation to comply with aid laws
		Occurrence of accidents in a field
		Not finding the accident site
		Impassable of path

### Core category

One of the main steps in conducting grounded theory studies is to identify the most important concerns of the participants. In this study, “uncertainty” was identified as the most important concern of the injured people, and “trying to save the injured person” was identified as the most important concern of the witnesses and families of the injured people. “Uncertainty” is the starting point of the process of trying to save the injured person. Based on the participants’ experiences, the uniqueness of accident, critical conditions, impatience and restlessness, pain and pressure, fear of losing a limb, and fear of subsequent accidents such as car explosion and patient death due to being stuck in the car would cause emotional behaviors, so that patients, their companions, and the people at the crash scene trying to solve the problem at any cost. On the other hand, previous experiences of the injured patient and their companions, seeing the heartbreaking scenes of the death of loved ones in the past and seeing the car explosion in the media after the accident cause people to get the injured person off the car with great effort. The critical situation of the injured person on the one hand and the spatial/environmental conditions, on the other hand, make the injured people, their companions, people at the crash site, and the emergency staff such as traffic police, who do not have sufficient knowledge on how to save people from a damaged car, take responsibility of the injured people and transfer them to hospital.

### Theoretical categories

#### Emotional interaction

In this category, four concepts explained emotional interaction in the crash site, which included “unplanned interventions,” “emotional behavior,” “emotional decision,” and “emotional intervention.” Emotional interaction means that interventions and decisions made by the companions of the injured people and witnesses of the accident are emotional and not only do not benefit the patient but also lead to complications such as secondary injury. One of the patients with spinal cord injury in this regard stated:

“They laid me down on the ground waiting for the ambulance to arrive, but because they thought my condition was critical, they took me to the side of the road, put me in a taxi, and drove me to hospital” (C. 6).

#### The scene shock

In the crash site, which is a unique scene, the injured people and their companions often fall into a state of instability and emptiness, which was identified as “the scene shock” in this study. For the scene shock, three concepts were developed: “Rescue efforts,” “Desperation,” and “Emotional atmosphere.”

One of the participants, who was with the injured person at the crash site, stated:

“At that moment, my mom said her hands and feet were getting cold and she could not feel them. I was very anxious and had a bad feeling. I was constantly thinking about an accident scene that had previously killed my uncle. I thought my mom is going to die too” (H. 4).

Another participant who was injured in an accident reported:

“My husband was crying and he was restless. It was very hard for him. Imagine you lose everything in an instant and can do nothing about it” (C. 7).

#### Insufficient understanding of the injury

This category was also associated with three concepts of “inadequate knowledge of emergency,” “lack of basic medical knowledge,” and “lack of comprehensive assessment.” These factors make people at the crash site not pay attention to the severity of spinal cord injury. Inadequate knowledge of emergencies and lack of training on the principles of first aid are among the factors that show insufficient understanding of spinal cord injury and ultimately not prioritizing the injury at the crash site. Lack of knowledge about the basics of patient transfer by witnesses and patients’ relatives increases the risk of spinal cord injury in patients suspected of having a spinal fracture. One of the injured patients in this regard stated:

“When the car crashed and people came to help, I was stuck in the car but could move my legs while the roof of my car was near my face. Someone came and took my hand, telling me to come out, you are not injured. I did not think my cervical vertebra was broken” (C. 2).

Another participant stated “They laid me down on the ground waiting for the ambulance to arrive, but because they thought my condition was critical, they took me to the side of the road, put me in a taxi, and drove me to hospital” (C. 6).

#### Misunderstanding of the delay

One of the underlying concepts was “the misunderstanding of the delay,” which was mentioned in all interviews. The belief of the injured person, people at the crash site, and even the emergency staff, such as traffic police, about the delay of emergency services, was one of the main reasons for the transfer of the injured person before the arrival of emergency services. The three concepts of “previous experiences of companions,” “accident shock,” and “lack of timely arrival of emergency services” explained the category of misunderstanding of the delay. The injured person’s restlessness and experience of observing injured people dying in accidents had created this mentality in the patients and their companions that there would be a possibility of death at any moment and therefore the injured person should be transferred to the hospital as soon as possible.

In this regard, one of the participants stated “I was very anxious, felt very bad, and had seen a past accident that resulted in the death of an injured person in front of my eyes. I was very stressed and anxious. I was calling the emergency service every minute and waited for someone to come and help and do something” (H. 4).

Having witnessed the death of loved ones at the crash site increases stress and anxiety and makes the transfer of a patient to the hospital the first and only priority. In the meantime, the belief in the late arrival of the emergency service reinforces this belief. One of the participants in this regard stated “Yes, in one case, the car was overturned. The injured person inside the car was bleeding and had multiple fractures. The car could not be moved. We moved the injured person out of the care, laid him down on the back seat of a car, and told the driver to take him to the hospital as quickly as possible. You know, every time we call the emergency service, they arrive late. They say they have only one ambulance and have to take care of multiple accidents” (P. 1).

### The study theory

A theory should be able to match all available observations and make accurate predictions about future observations. Theories are patterns of real-world phenomena that determine the components or elements of a phenomenon and the relationships between them. Theories are made up of hypotheses that are necessarily based on empirical data (77). In this study, the theory of “uncertainty in traffic accidents” is as follows:

According to studies, when traffic accidents occur and lead to spinal cord injury, uncertainty is the main concern of all those present at the crash site. This concern causes reactions such as delegation of responsibility, emotional participation, and escape from the crash scene, which is intended to save the injured person. Trying to save the injured person, inadequate emergency services, insufficient understanding of the injury, inability to manage the injury or secondary injury to a patient, and various cultural, social, and political factors such as misunderstanding of the delay as well as lack of system support, heroic behaviors, and cultural beliefs are among the factors that influence the concept of “uncertainty.”

## Discussion

This qualitative study was conducted using the grounded theory approach and Corbin and Strauss’s method (17). This study aimed to design a model for spinal cord injury due to road traffic accident in the pre-hospital scene. Data analysis yielded five main categories, including emotional participation, scene shock, misunderstanding of the delay, delegation of responsibility, and insufficient understanding of the injury. These categories indicated that when a traffic accident occurs, everyone on the crash site, from pre-hospital emergency staff, traffic police, and law enforcement officers to the patient’s companions and witnesses of the accident, tries to save the injured people.

Uncertainty, which is their main concern and anxiety, shows itself through excessive anxiety and despair caused by the shock of the incident, which is the first reaction of the audience. Therefore, the patient’s companions and the witnesses begin to intervene, which is a kind of reaction to the situation to save the life of the injured people. Another group of crash witnesses delegates their responsibility of assisting others and refuse to assist due to the fear of the injured person’s unstable conditions, fear of secondary injuries to the injured, self-harm, following others’ instructions, and cultural barriers. Cultural and philanthropic beliefs, misunderstandings of the delay, heroic behavior, stress, lack of coordination between rescue workforces, and lack of system support for pre-hospital emergencies are the underlying factors that contribute to spinal cord injury in traffic accidents. The consequences of these factors include insufficient understanding of the injury, the inadequacy of emergency service, inability to manage the injury, and most importantly, infliction of secondary injury to the injured people.

The concept of “uncertainty” in the present study was the main concern of the injured people, their companions, and witnesses of the accident. Uncertainty is a human response to environmental stimuli and a dynamic situation in which it is improbable to predict or determine the consequences of an event. Veismoradi stated that uncertainty is managed by a sense of self-confidence and self-control. Uncertainty occurs in situations where there is a lack of available evidence, differences in the interpretation of an incident, or disagreement over available evidence (19). In line with the results of the present study, Forslund (20) investigated the challenges of pre-hospital care from the perspective of casualties, companions, and emergency technicians and found that life-threatening conditions in emergencies and the unpredictable and unique nature of these situations are associated with the sense of loneliness, uncertainty, and dependence (20). In another study, Prati stated that when people are exposed to an emergency, their first reaction is to escape from the scene. This reaction is a common reaction to fear and insecurity, which are seen when the person does not have the power and capacity to manage the situation (21). In the study of Grimm (22) on the behaviors and emotions of people exposed to life-threatening accidents, the most common responses of people were the fear of perceived danger and the struggle to survive. To save their lives, people took actions that were considered wrong. One of these actions was to escape from an unsafe place (22). Erlanson et al. (23) showed that police officers were experiencing a sense of insecurity and uncertainty at the crash site, especially when they did not know how to effectively manage the injured people.

The scene shock was one of the sub-concepts related to “uncertainty.” This concept can also be found in several studies, including the studies of Khankeh et al. and Nakhaei et al. (24, 25). The scene shock refers to a situation in which the injured people and their companions fall into a state of instability and emptiness, and psychological pressures prevent companions to make a logical and rational decision. The witnesses of the accident and companions of the injured people, who observe the unstable conditions of the injured people, often start screaming and crying. Being in a situation that has not been experienced before and having a sense of perceived death would cause people to feel confused and helpless. The injured person’s restlessness and request for help increase the sense of helplessness. Lack of knowledge on how to manage the injured person increases the anxiety of people at the crash site.

In a grounded theory study conducted by Nakhaei et al. (25) on life after accidents and disasters, one of the concepts extracted from the study was the shock of exposure to the accident scene. This means that accident is associated with personal and financial injuries and imposes a high level of stress and psychological pressure on people. This shock affects people’s responses and behaviors, causing them to take ineffective actions (25). Stress, pressure, and excitement make the patient’s companions make an effort to save the patient’s life when the situation is unstable and there is a possibility of another accident such as a car explosion. This action results from the scene shock and the emotional reactions of those around. In a study of spinal cord injury after an earthquake, Priebe stated that after experiencing a shock in the early minutes of the earthquake, those who acted as lifeguards at the scene trying to save the lives of the injured people were often unaware of the fact that people who are suspected of spinal cord injury should only be moved if their lumbar spine and neck are fixed (26). As a result, they were dragging the injured on the ground away from the danger without fixing their spine.

In this study, the participants used different strategies to solve the problem and save the injured person. One of these strategies used by the companions and witnesses of the accident was “emotional interaction.” The concept of emotional interaction was one of the main categories extracted from the present study. It refers to the fact that people who witness the accident are usually the first to appear at the crash site. The presence of people at the crash site is one of the important pillars that separates the nature of pre-hospital services in traffic accidents from other emergencies. At the scene of an accident, those who are nearby as well as the injured people’s companions often perform hasty actions and unnecessary interventions such as the transfer of patients due to the lack of knowledge about the patient transfer as well as a sense of helplessness. Unprotected removal of the injured person from the car, improper manual handling of the injured person, rushed patient transfer, and non-stabilization of the injured person are among the inappropriate actions and interventions taken by people at the crash site.

According to the studies of Alinia and Khorasani, performing incorrect activities and inappropriate interventions aggravate injuries. Anxiety and stress at the crash site are one of the factors that cause people and companions of the injured people to take hasty actions (27, 28). In this regard, Khorasani Zavareh showed that people arrived at the crash site earlier than the emergency personnel and often had a high level of stress and anxiety. They also interfered with the work of emergency personnel and sometimes caused secondary injuries to the injured and their companions or even their death (28). In the present study, the companions of the injured people at the crash site were emphasizing the rapid transfer of the injured people to the hospital, and for this reason, the injured people were usually removed from the car and transferred to the hospital by private vehicles. In a similar study in India, Pallavisarji et al. found that public interventions for victims of traffic accidents included contacting emergency services and transferring injured people to hospitals by private vehicles (29). The emotional performance of laypeople at the crash site, which is due to the emotional atmosphere and the hasty attempt to save the injured person, often causes secondary and unintended injuries to the injured people.

Another component of emotional participation is emotional behavior, which is often associated with emotional feeling and even in some cases confrontation with the emergency personnel. Emotional behavior is one of the reactions of people at the crash site, which manifests itself in the form of violence against the emergency personnel, insisting on quick rescue and transfer of the injured people to the hospital. This emotional feeling often makes people remove the injured people from the vehicle, which aggravates the spinal cord injury. According to studies conducted in Iran, the emotional reactions of laypeople at the crash site along with anger and verbal abuse of emergency personnel are the result of people’s presence at the crash site (30, 31). Poursheikhian argues that severe injuries to injured people cause anxiety and restlessness in the patient’s companions and others, resulting in uncontrolled and unpredictable behaviors such as violence and aggression (30). On the other hand, anxiety and despair have other consequences, as in the systematic review conducted by Heidari et al. (32), sometimes the confusion and anxiety of laypeople at the crash site led to multiple requests for an ambulance (even more than what was required), a crowded scene, and disruption in the work of emergency personnel at the crash site (32). These findings are in line with the results of the present study. Making immediate and unplanned decisions is the result of emotional behaviors that take place in the context of the prevailing emotional atmosphere. This emotional decision is the result of unfavorable conditions, bleeding, and multiple fractures of the injured people. A lack of knowledge about the correct principles of triage and injury assessment causes people at the crash site to consider the situation critical and make a hasty decision. In the study of Haghparast et al., people’s lack of knowledge about the principles of first aid at the crash site was one of the reasons that led to ineffective care for the injured people (15).

Another finding of this study was the misunderstanding of the delay. Witnesses at the crash site in this study participated in the transfer of the injured people to a hospital even before the arrival of pre-hospital emergency service, citing the belief that the emergency service was certainly going to have a delay. Obstacles such as traffic caused by the accumulation of private cars near the crash scene or the remote location of the road often have negative consequences. This delay not only causes the injured to be taken to the hospital late but also in some cases intensifies the excitement of people at the crash site, engraving the belief that the emergency services are always late. In this regard, Alinia et al. (27) reported traffic and the difficulty of urban roads were among the factors that led to the delay of pre-hospital emergency services. In a grounded theory study, Pourshekhian (30) stated that delay in response times is due to traffic, inability to use GPS, difficulty finding addresses, and problems in the communication system, which lead to restlessness, anxiety, and violence against emergency personnel, or transfer of patient before the arrival of emergency services.

Haghparast, Khorasani, and Bigdeli also highlighted the traffic as an obstacle to achieving the desired emergency response time (10, 33, 34). Alinia et al. indicated that sometimes pre-hospital emergency personnel has to stop the ambulance away from the scene and walk a short distance due to the presence of too many people at the crash site. This factor not only delays the delivery of aid but also in some cases leads to errors in estimating the response time because such stops are not calculated in the response timing (27). Previous experiences of companions and witnesses of traffic accidents about the delay of emergency services can strengthen this belief in them, causing them to intervene in the next accident before the arrival of emergency personnel.

Another consequence of uncertainty in traffic accidents was an inadequate understanding of the injury. According to the participants, lack of training on the principles of first aid was also one of the factors at the crash site that showed insufficient understanding of spinal cord injury and ultimately not prioritizing the injury by people. Lack of knowledge about the basics of manual handling by witnesses of a traffic accident and companions of the patient increases the risk of spinal cord injury in patients suspected of vertebral fracture. Further, paying too much attention to visible injuries (such as open fractures and external bleeding) and managing them can distract people at the crash site from considering the main injuries, such as spinal cord injury. Confirming the above findings, Haghparast et al. stated that a lack of knowledge about pre-hospital care and first aid and also a lack of public education programs make people adopt measures that have negative effects (10).

Furthermore, Khorasani Zavareh et al. (34) evaluated the barriers and facilitators of post-accident injury management and showed that one of the most important barriers in the post-accident phase was the interaction of untrained people and their lack of knowledge about managing the situation and the injured people. Training target groups, including vehicle drivers, high school students, soldiers, and volunteers, is one of the most effective ways to improve pre-hospital care. In a study, Priebe stated that first-time witnesses, who are generally laypeople, should be educated about the importance of proper patient handling to prevent neurological damage (26). Nakhaei et al. (25) argued that one of the causes of secondary injuries to patients after the Bam earthquake was the intervention of untrained people who had transferred the injured to a hospital without having standard operating protocols. Lack of knowledge about the anatomy and physiology of the spinal cord and its function causes people at the crash site not to pay attention to spinal cord injury (35). Since spinal cord injury has no obvious signs and symptoms such as bleeding, often no one pays attention to it. In addition, the unstable condition of the injured people and the stress of people at the crash site do not allow them to analyze the scene and consider internal injuries such as spinal cord injuries (36).

In the present study, some injured subjects with SCIs were not interviewed due to stress. This is because of the occurrence of flashbacks and being drawn back into the traumatic experiences. Furthermore, most of the interviews were conducted in the hospital and rehabilitation centers; therefore, the patients had to undertake occupational therapy and physiotherapy. This was one of the issues that could interfere with the interview process. In such situations, the interviewer had to continue the interview after performing the rehabilitation activities; however, some patients were unable to continue the interview or focus due to fatigue.

## Conclusion

The present study was taken the form of a doctoral dissertation conducted using a grounded theory approach. The main question of this study focused on how spinal cord injury is caused in traffic accidents and identifying the factors and processes involved in causing spinal cord injury. Relying on the lived experiences of the participants, this study clarified the concerns of people and how people interact and respond to traffic accidents from the perspective of the injured, their familes, and laypeople (witnesses of the traffic accident). According to the findings, in a traffic accident, uncertainty about the situation is the main concern of everyone at the crash site, from pre-hospital emergency personnel, traffic police, and law enforcement officers to the patient’s familes and other witnesses. Uncertainty will increase in the next incidents, which means that people will look at their previous experience and this mindset will be created that the interventions of different aid organizations in this process will increase the damage. Therefore, this belief is strengthened and the uncertainty becomes more and heavier in the next traffic accidents. By teaching, it is possible to manage the accident scene to a great extent and minimize the negative consequences of the accident.

## Data Availability

The raw data supporting the conclusions of this article will be made available by the authors, without undue reservation.
